# Time to Align: A Call for Consensus on the Assessment of Genetic Testing

**DOI:** 10.3389/fpubh.2021.807695

**Published:** 2021-12-06

**Authors:** Erica Pitini, Valentina Baccolini, Giuseppe Migliara, Claudia Isonne, Alessandro Sindoni, Elena Mazzalai, Federica Turatto, Corrado De Vito, Carolina Marzuillo, Paolo Villari

**Affiliations:** Department of Public Health and Infectious Diseases, Sapienza University of Rome, Rome, Italy

**Keywords:** genetic testing, genetic test, genomic test, EUnetHTA, public health genomics, Health Technology Assessment

## Abstract

In this paper, we updated our 2018 systematic review aimed to identify and compare *ad hoc* designed frameworks for genetic testing evaluation. Overall, we identified 30 frameworks (29 in the first systematic review and one in the update): they were mainly based on the ACCE model, whereas a minority were adjustments of the more traditional Health Technology Assessment (HTA) approach. After discussing the strengths and weaknesses of the retrieved frameworks, this perspective calls for consensus on the assessment of genetic testing. In line with the recent European recommendations that encouraged the generation of comparable evidence across Member States, we believe that the time has come to align all the ideas that have emerged over the last few decades and find a sustainable and sharable tool for the evaluation of genetic and genomic applications. Therefore, we suggest stopping the evaluation of such technologies using *ad hoc* strategies–affected by validation, implementation, and adoption issues–and we propose to use a general HTA approach, particularly the European reference tool for the assessment of health technologies, the EUnetHTA HTA core model, that is built on solid theoretical and methodological principles and provides a comprehensive assessment of the technologies value.

## Introduction

The assessment of the risk and benefits of genetic and genomic tests has long been addressed using *ad hoc* evaluation methods, as we described in a previous systematic review of such evaluation frameworks created over the last decades (1). The majority of the 29 frameworks reviewed were based on the ACCE model, which was specifically designed for genetic tests and whose name derives from the evaluation dimensions used, i.e., Analytic validity, Clinical validity, Clinical utility, Ethical, legal, and social implications. Since its launch in the early 2000s by the United States (US) Centers for Disease Control and Prevention, the ACCE model has imposed its own dimensions and terminology on the assessment of genetic and genomic tests (2). The main alternatives to the ACCE model were based, instead, on adjustments of the more traditional Health Technology Assessment (HTA) approach, which was established in the US in the late 60s as a general evaluation framework intended to cover all health technologies.

## Strengths and Weaknesses of the Available Frameworks for the Assessment of Genetic and Genomic Technologies

The ACCE model examines in detail the technical aspects of genetic and genomic tests, particularly their analytic validity and clinical validity, which need to be understood before their clinical efficacy can be assessed. On the other side, HTA-based frameworks allow a more systematic analysis of the economic and organizational aspects of the delivery of the genetic testing programme as a whole; this is important, particularly for universal healthcare systems, as it allows an efficient and equitable allocation of healthcare resources (1).

To combine the best aspects of the ACCE model and the HTA process, we published in 2019 a proposal for an integrated framework aimed at assessing both the genetic test and its delivery models (3). In this new framework, the assessment of the technical and clinical value of a genetic test is mainly based on the ACCE evaluation dimensions (i.e., analytic validity, clinical validity, and clinical utility), with the single addition of the personal utility dimension, i.e., an assessment of the non-clinical outcomes that the test may exert on patients. On the other hand, the assessment of the genetic testing delivery models uses the HTA approach, adopting the relevant evaluation dimensions (organizational aspects, economic evaluation, ELSI, and patient perspective) of the EUnetHTA HTA core model, which is the European reference tool for the assessment of health technologies (4).

While this new framework has the advantage of integrating genetic test-specific and widely recognized evaluation dimensions and terminology within a traditional HTA approach, its extensive adoption is undermined by fundamental weaknesses. It has not yet been adequately validated and, in particular, it is difficult for it to compete with more established frameworks, such as the EUnetHTA HTA core model itself. This latter model has been developed and piloted according to rigorous research protocols by the European network for HTA, which involves more than 80 institutions in 29 European countries; it is available in several applications each of which focuses on a different type of technology, and it is continuously under revision to produce updated versions (5, 6).

**Figure 1:**
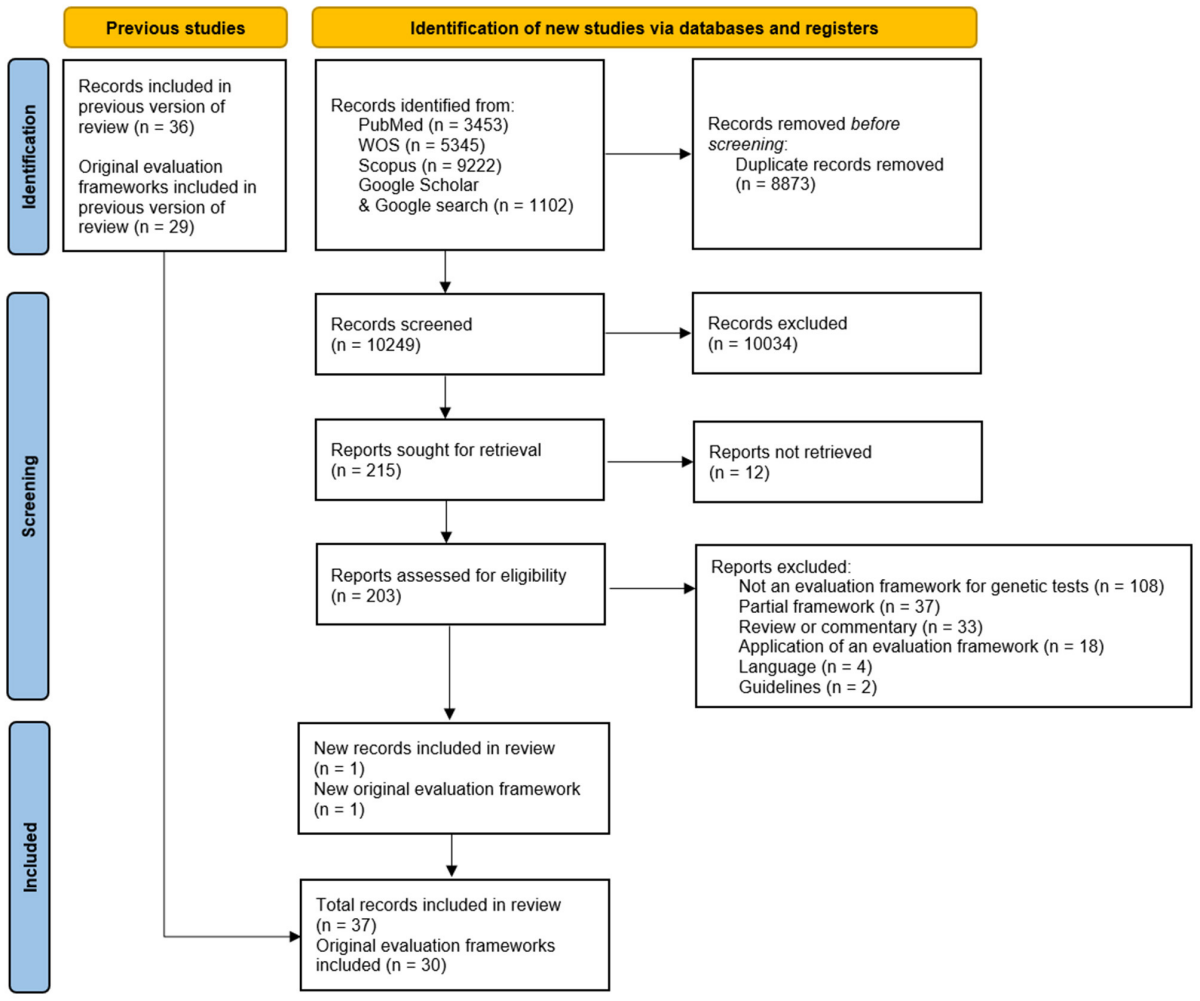
PRISMA 2020 flow diagram for the 2021 update of our 2018 systematic review.

To be fair, the same limitations of our framework (validation, implementation, adoption) also apply to a significant proportion of the frameworks retrieved by our systematic review. The good news is that the proliferation of theoretical models for the assessment of genetic and genomic tests over the last 20 years seems to have come to an end. In fact, we have just performed an update of our 2018 systematic review using the same methodology, but the only new framework returned was ours (Figure 1).

## European Recommendations

In 2018, the European Commission adopted a legislative proposal to boost cooperation amongst EU Member States when assessing health technologies, and to promote convergence toward the use of HTA tools, procedures and methodologies already developed by the EUnetHTA Joint Actions (7). Furthermore, in the same year, cooperation was also advocated in the specific field of genomics with the “1 + Million Genomes” (1 + MG) initiative, which now involves 24 European countries (8). As part of this initiative, the generation of comparable evidence across European countries was strongly encouraged; this is expected to facilitate policy choices that translate genomic innovation into effective and cost-effective healthcare, as well as improving the sharing of results (9).

## Discussion

Finding a sustainable, shared path toward a universal evaluation framework for genetic and genomic tests is still a priority. In this regard, we believe the time has come to align all the ideas that have emerged over the last few decades and to reach a consensus on an evaluation framework that will guide the evaluation process and maximize population health benefits across Europe and more globally. This requires that genetic and genomic tests are no longer assessed using *ad hoc* strategies, but instead are evaluated by a general HTA approach that employs a common methodology, but is nevertheless capable of addressing the individual characteristics of each test. Thus, we suggest taking as a reference the appropriate applications of the EUnetHTA HTA core model and considering whether these should be used “as is” or whether they should be integrated with specific content when the technology under assessment is a genetic and genomic test. In this way, we could have a reference tool for the evaluation of genetic tests built on solid theoretical and methodological principles, entirely (or almost entirely) validated, capable of a comprehensive assessment of all the technical, clinical and delivery aspects and, last but not least, commonly shared across Europe.

## Data Availability Statement

The raw data supporting the conclusions of this article will be made available by the authors, without undue reservation.

## Author Contributions

EP, VB, GM, and PV contributed to conception and design of the study. CI, AS, EM, and FT performed the update of the systematic review (bibliographic search and data extraction). EP and GM contributed to data curation. EP wrote the first draft of the manuscript. VB wrote sections of the manuscript. PV, CM, and CD contributed to supervision and funding acquisition. All authors contributed to manuscript revision, read, and approved the submitted version.

## Funding

This work was supported by the Italian project Definizione e promozione di programmi per l'implementazione delle azioni centrali di supporto al Piano per l'innovazione del sistema sanitario basata sulle scienze omiche (Definition and promotion of programs to support the implementation of the Italian Omics science Plan), funded by the Italian Ministry of Health–National Centre for Disease Prevention and Control.

## Conflict of Interest

The authors declare that the research was conducted in the absence of any commercial or financial relationships that could be construed as a potential conflict of interest.

## Publisher's Note

All claims expressed in this article are solely those of the authors and do not necessarily represent those of their affiliated organizations, or those of the publisher, the editors and the reviewers. Any product that may be evaluated in this article, or claim that may be made by its manufacturer, is not guaranteed or endorsed by the publisher.
